# *Pseudomonas aeruginosa* Affects Airway Epithelial Response and Barrier Function During Rhinovirus Infection

**DOI:** 10.3389/fcimb.2022.846828

**Published:** 2022-02-21

**Authors:** Adrian Endres, Christian Hügel, Helena Boland, Michael Hogardt, Ralf Schubert, Danny Jonigk, Peter Braubach, Gernot Rohde, Carla Bellinghausen

**Affiliations:** ^1^ Department of Respiratory Medicine and Allergology, University Hospital Frankfurt, Goethe University, Frankfurt am Main, Germany; ^2^ Consiliary Laboratory on Cystic Fibrosis Bacteriology, Institute of Medical Microbiology and Infection Control, University Hospital Frankfurt, Goethe University, Frankfurt am Main, Germany; ^3^ Department for Children and Adolescents, University Hospital Frankfurt, Goethe University, Frankfurt am Main, Germany; ^4^ Institute for Pathology, Hannover Medical School, Hannover, Germany; ^5^ German Center for Lung Research (DZL), Biomedical Research in End-stage and Obstructive Lung Disease Hannover (BREATH), Hannover, Germany

**Keywords:** co-infection, cystic fibrosis, bronchial epithelium, respiratory infections, rhinovirus, *Pseudomonas aeruginosa*

## Abstract

Cystic fibrosis (CF) lung disease is aggravated by recurrent and ultimately chronic bacterial infections. One of the key pathogens in adult CF lung disease is *P. aeruginosa* (PA). In addition to bacteria, respiratory viral infections are suggested to trigger pulmonary exacerbations in CF. To date, little is known on how chronic infections with PA influence susceptibility and response to viral infection. We investigated the interactions between PA, human rhinovirus (HRV) and the airway epithelium in a model of chronic PA infection using differentiated primary bronchial epithelial cells (pBECs) and clinical PA isolates obtained from the respiratory sample of a CF patient. Cells were repeatedly infected with either a mucoid or a non-mucoid PA isolate for 16 days to simulate chronic infection, and subsequently co-infected with HRV. Key cytokines and viral RNA were quantified by cytometric bead array, ELISA and qPCR. Proteolytic degradation of IL-6 was analyzed by Western Blots. Barrier function was assessed by permeability tests and transepithelial electric resistance measurements. Virus infection stimulated the production of inflammatory and antiviral mediators, including interleukin (IL)-6, CXCL-8, tumor necrosis factor (TNF)-α, and type I/III interferons. Co-infection with a non-mucoid PA isolate increased IL-1β protein concentrations (28.88 pg/ml vs. 6.10 pg/ml), but in contrast drastically diminished levels of IL-6 protein (53.17 pg/ml vs. 2301.33 pg/ml) compared to virus infection alone. Conditioned medium obtained from co-infections with a non-mucoid PA isolate and HRV was able to rapidly degrade recombinant IL-6 in a serine protease-dependent manner, whereas medium from individual infections or co-infections with a mucoid isolate had no such effect. After co-infection with HRV and the non-mucoid PA isolate, we detected lower mRNA levels of Forkhead box J1 (FOXJ1) and Cilia Apical Structure Protein (SNTN), markers of epithelial cell differentiation to ciliated cells. Moreover, epithelial permeability was increased and barrier function compromised compared to single infections. These data show that PA infection can influence the response of bronchial epithelial cells to viral infection. Altered innate immune responses and compromised epithelial barrier function may contribute to an aggravated course of viral infection in PA-infected airways.

## Introduction

In cystic fibrosis (CF), lung disease is the main contributor to morbidity and mortality. It is characterized by impaired mucociliary clearance, excessive inflammation and destruction of lung tissue (Bergeron and Cantin, 2019). CF lung disease is accompanied by frequent and ultimately chronic bacterial infection in most adult patients. A key pathogen in CF lung disease is *Pseudomonas aeruginosa* (PA). Chronic infections with this pathogen are associated with accelerated disease progression, recurrent exacerbations and worsened health status (Malhotra et al., 2019).

PA is a versatile microorganism that can be found in respiratory specimens of CF patients in different phenotypes. Acute infections are mostly linked to a PA phenotype characterized by non-mucoid colony morphology, whereas during chronic infections, PA frequently convert to a mucoid phenotype. Mucoid PA are characterized by overproduction of the exopolysaccharide alginate [reviewed, for example, by Hogardt and Heesemann (2010)], which additionally hampers eradication by the immune system and antibiotic treatment. In chronic lung infections in CF, mucoid and non-mucoid phenotypes are often isolated simultaneously from respiratory secretions (Malhotra et al., 2019).

Although it is known that chronic infection with PA negatively impacts health status and disease progression in CF (Garcia-Clemente et al., 2020), our knowledge of its interplay with viral infection remains limited. The relevance of respiratory viruses in the course of CF lung disease is increasingly recognized, as viral infections have been found to be associated with an increased risk for pulmonary exacerbations and increased respiratory symptoms in patients with CF (Flight et al., 2014).

There is growing evidence that preceding bacterial colonization and infection can alter the course and outcome of subsequent viral infection (Sajjan et al., 2006; Ichinohe et al., 2011; Wang et al., 2013; Wolf et al., 2014a; Wolf et al., 2014b; Gulraiz et al., 2015; de Steenhuijsen Piters et al., 2016; Ederveen et al., 2018; Sonawane et al., 2019). As the majority of adult CF patients suffers from chronic bacterial pulmonary infections, this could have severe clinical implications for many CF patients.

Mechanistically, interactions between bacterial infections and viral pathogens relevant in CF lung disease are insufficiently understood. The epithelium of the respiratory tract plays a pivotal role in the defense against respiratory pathogens, not only by constituting a tight physical barrier, but also through mucociliary clearance, recognition of pathogen-associated molecular patterns and coordination of early innate immunity. Epithelial cell-derived cytokines contribute to the recruitment and activation of specialized immune cells. Moreover, epithelial cells themselves can produce antiviral and antibacterial mediators that directly contribute to the control of respiratory infections (Vareille et al., 2011; Hiemstra et al., 2015). Being the major target for most respiratory viruses and contact surface for colonizing bacteria, epithelial cells are moreover a crucial interface in the interaction between viruses, bacteria, and the host.

To investigate the effects of PA infection on epithelial antiviral responses in CF and other diseases, we established a chronic PA infection model of differentiated primary bronchial epithelial cells. Using this model, we investigated the effects of *P. aeruginosa* isolates on cytokine production and epithelial barrier function during subsequent infection with human rhinovirus.

## Materials and Methods

### Cell Culture

Primary bronchial epithelial cells (pBECs) were isolated from lung explant tissue of three CF and three lung emphysema patients undergoing lung transplantation. The tissue was obtained from the Hannover Pathology Tissue collection (Hannover Medical School) and its use was approved by the institutional ethics review board of Hannover Medical School (Reference 2700-2015). Donor characteristics are shown in **Supplementary Table S1**.

Cells were isolated using a protocol modified from van Wetering et al. (2000). One to two bronchus rings were used for each isolation. In order to detach cells from the bronchus, the bronchial ring was cut open and incubated for 2 h at 37°C in protease XIV solution (final concentration 1.8 mg/ml, Sigma, St. Louis). It was then transferred to a sterile Petri dish containing PBS supplemented with antibiotics (penicillin/streptomycin, Sigma, St. Louis; MycoZap Plus PR, Lonza, Basel), and cells were detached by gently scraping the inner side of the bronchial ring. Cells were pelleted by centrifugation, resuspended in KSFM complete medium (Gibco, Carlsbad) supplemented with 25 µg/ml bovine pituitary extract (Thermo Fisher Scientific, Waltham), 0.2 ng/ml epidermal growth factor (Thermo Fisher Scientific, Waltham), 1 nM isoproterenol (Sigma, St. Louis), penicillin/streptomycin and MycoZap, and seeded in tissue culture flasks pre-coated with 10 µg/ml fibronectin (VWR, Radnor), 10 µg/ml BSA (Sigma, St. Louis) and 30 µg/ml PureCol collagen (Sigma, St. Louis). Culture medium was replaced every other day until cell layers reached approximately 80% confluence. Cells were then cryopreserved at a controlled freezing rate in KSFM with 0.3 mg/ml BPE and 10% DMSO and stored in liquid nitrogen until further use.

Isolated cells were confirmed to be basal epithelial cells by immunofluorescence. Cells stained positive for cytokeratin 5 (rabbit anti-human cytokeratin 5, Biolegend), p63 (rabbit anti-human p63, Abcam, Cambridge) and negative with the fibroblast-reactive antibody TE-7 (mouse anti-human fibroblast antigen, clone TE7, Merck, Darmstadt).

### Culture of pBECs as Air-Liquid Interface Cultures

For each experiment, cells were thawed from cryopreserved stocks and expanded in pre-coated tissue culture flasks using PneumaCult Ex medium including supplements (Stemcell Technologies, Vancouver). Cells were passaged once before seeding into the apical chamber of 12-well plates with transwell inserts at 4x10^5^ cells/insert. Until cell layers were fully confluent, cells were cultured as submerged cultures with PneumaCult Ex medium on both, apical and basal side of the chamber, with medium being changed every other day. Once cell layers reached confluency, the medium on the apical side was removed and cells were left air exposed, while medium on the basal side was replaced with PneumaCult ALI medium including supplements (StemCell Technologies, Vancouver), penicillin/streptomycin (Sigma, St. Louis) and MycoZap Plus PR (Lonza, Basel) in order to initiate differentiation. Medium was refreshed and cells were washed with PBS three times a week until cell layers were fully differentiated after approximately three to four weeks. Cell differentiation was confirmed by an increase in transepithelial electric resistance (TEER), mucus production, and microscopically visible ciliary movement. At least one week prior to infection, cultures were transferred to antibiotic-free medium.

### Bacteria and Viruses

Two *P. aeruginosa* (PA) isolates, one with non-mucoid phenotype and one with mucoid phenotype, were recovered from one respiratory sample of a CF patient with chronic PA lung infection by using CF-specific cultivation conditions including MacConkey and *Pseudomonas* CFC selective agar (ThermoFisher, Waltham). Phenotypes were assigned based on colony morphology (**Supplementary Figure 1**). Species identification was done by Matrix-assisted-laser desorption ionization-time of flight analysis (MALDI-TOF; VITEK MS; bioMérieux, Nürtingen). Subcultures were picked from single colonies and cryopreserved as pure cultures in Microbank vials (Pro-Lab Diagnostics, Round Rock).

For experiments, bacterial suspensions were prepared in PBS from bacteria grown overnight on Columbia Agar plus Sheep Blood ‘Plus’ plates (ThermoFisher Scientific, Waltham). To achieve consistent bacterial inocula, bacterial suspensions were first diluted to an OD_600_ of 0.4 and then further diluted to achieve the desired colony count using a strain-specific dilution factor determined experimentally beforehand.

Human Rhinovirus (HRV) type 16 was originally obtained from ATCC (Manassas) and propagated in HeLa Ohio cells (Sigma Aldrich, St. Louis). For preparation of stocks, sub-confluent cell layers were inoculated with the parent virus pool at a multiplicity of infection of approximately 0.1 in DMEM + 2% fetal bovine serum (FBS). Virus was allowed to attach for 2 h at 33°C under occasional gentle swirling. Unbound virus was then removed by washing cells twice with PBS and fresh infection medium (DMEM+ 2% FBS) was added to the flasks. Approximately three days after infection and when 80-90% of the cell layer showed cytopathic effect, virus pools were harvested. Culture supernatants were collected and cleared of cell debris by centrifugation. Additionally, remaining cells were detached with a cell scraper in a small volume of medium, snap frozen in liquid nitrogen, thawed and centrifuged to remove cell debris. The supernatant of this centrifugation step was added to the cleared culture supernatant. Virus pools were concentrated 10-20x and partially purified by ultrafiltration using Amicon Ultra Centrifugal filter units (cutoff 100 kDa). Virus stocks were quantified by determining the 50% Tissue Culture Infectious Dose (TCID_50_) on HeLa Ohio cells. TCID_50_ was calculated using the Spearman-Karber Formula (Hierholzer and Killington, 1996).

### Chronic PA Infection Model

**Figure 1:**
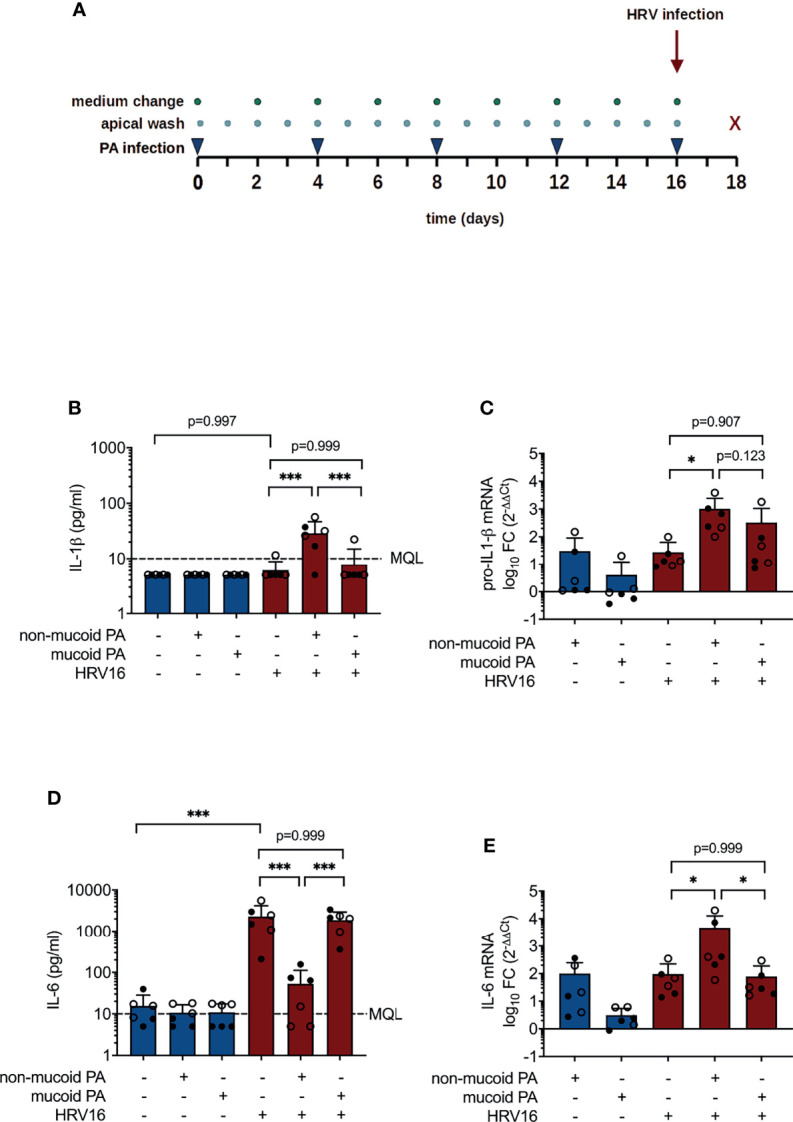
Cytokine production in differentiated bronchial epithelial cells co-infected with *P. aeruginosa* (PA) and human rhinovirus 16 (HRV16). **(A)** Infection protocol/timeline **(B)** IL-1ß protein concentrations in basal media **(C)** pro-IL-1ß mRNA levels **(D)** IL-6 protein concentrations in basal media **(E)** IL-6 mRNA levels. MQL, Minimum quantifiable level, FC, fold change. Cell type: ○ Emphysema ● Cystic fibrosis. Data were obtained in six independent experiments, each performed with cells derived from a different donor. *p ≤ 0.05, **p ≤ 0.01, ***p ≤ 0.001.

To simulate chronic bacterial infection, cells were repeatedly infected with PA for a period of 16 days (**Figure 1A**). For each infection, cell layers were washed thoroughly with PBS and 10^3^ colony forming units (CFU) of the respective PA strain were added to the apical side of inserts, diluted in 50 µl PBS with 25 µg/ml tobramycin. Cell layers were washed daily, and basal medium was changed every 48 h. On day 16, after apical washing, cells were infected with HRV16 from the apical site at a multiplicity of infection of 1. After incubation at 33°C for 2 h, bacterial infection was carried out as described above, followed by incubation for 48 h at 33°C.

Bacterial infections were carried out in the presence of tobramycin in order to prevent bacterial overgrowth and excessive cell death. Tobramycin was chosen due to its clinical relevance in the treatment of pulmonary PA infections in CF. Both PA isolates were sensitive to tobramycin (minimum inhibitory concentrations [MIC]: 4 µg/ml for non-mucoid isolate, 2 µg/ml for mucoid isolate as determined by E-test). Despite the used concentrations of tobramycin being above the respective MICs of the two isolates determined by E-test, live bacteria could be recovered and cultured from apical washes of the infected cell cultures.

### Reverse Transcription Quantitative PCR

Gene expression and viral RNA were quantified by RT-qPCR. Total RNA was isolated using a peqGOLD Total RNA Kit (VWR, Radnor) and 100 ng RNA was reverse transcribed using iScript gDNA Clear cDNA Synthesis Kit (BioRad, Hercules). For qPCR analysis, cDNA was diluted 1:5. qPCR was then performed using Sso Advanced Universal Probes Mastermix (BioRad, Hercules) on a StepOne Plus Real-Time PCR system (ThermoFisher, Waltham). Oligonucleotide sequences are listed in the **Supplementary Information (Table S2)**. For gene expression analysis, fold changes relative to uninfected controls were calculated using the 2^-ΔΔCT^ method (Livak and Schmittgen, 2001) with GAPDH as normalization gene. Intracellular viral RNA copies were quantified using a standard curve derived from synthetic double-stranded DNA.

### Determination of Cytokine Concentrations

Protein concentrations of IL-6 and CXCL-8 were quantified by ELISA (Invitrogen, ThermoFisher). Protein concentrations of IL-1β, TNF-α, CXCL-10, and TGF-β were determined by cytometric bead array (BD, Franklin Lakes).

### Degradation of Recombinant IL-6 by Conditioned Media

To test for potential degradation of IL-6, 10 µl conditioned culture medium obtained from infection experiments was used either untreated, heat-treated (95°C/5 min), or mixed with 8 µl of different protease inhibitors (or respective controls) and then incubated with 2 µl (0.5 µg/µl) recombinant IL-6 (Peprotech, Rocky Hill) at 37°C. For inhibition of metalloproteases, phosphoramidon (Sigma-Aldrich) was used at a final concentration of 3.4 mM. Nα-Tosyl-L-lysine chloromethyl ketone hydrochloride (TLCK, Sigma-Aldrich), primarily inhibiting serine proteases, was used at a final concentration of 40 mM. Additionally, a proprietary, broad non-metalloprotease inhibitor was used (cOmplete™ Mini EDTA-free Protease Inhibitor Cocktail, Roche, one tab dissolved in 500 µl water).

### Western Blot

Proteins were separated by sodium dodecyl sulfate (SDS)–polyacrylamide gel electrophoresis using a 15% polyacrylamide gel and transferred onto a nitrocellulose membrane. The membrane was then blocked with 5% skim milk for 1 h at room temperature and subsequently incubated with mouse anti-human IL-6 monoclonal antibody (Clone OTI3G9, Origene, Rockville) diluted 1:50000 in 5% skim milk. After washing with TBST, the membrane was incubated with an HRP-conjugated secondary goat anti-mouse antibody (Thermo Fisher Scientific, Waltham, dilution 1:5000) for 1 h at ambient temperature. After thorough washing of the membrane, signals were detected using Western-Blot Detection Reagents (Amersham/GE Healthcare, Little Chalfont) according to the manufacturer’s protocol. Densitometry was performed for the peak corresponding to full length IL-6 using FIJI/ImageJ2.

### Assessment of Epithelial Barrier Function

Transepithelial electrical resistance (TEER) was measured directly prior to and 48 h after (co-)infection (Millicell ERS-2 instrument, MerckMillipore, Burlington). To measure permeability of the cell layer, 100 µl FITC-labeled dextran (5 mg/ml, average molecular weight 20 kDa, Sigma-Aldrich) were added to the apical side 48 h after (co-)infection. After one hour, fluorescence was measured in medium taken from the basal side (EnVision Multimode Plate Reader, PerkinElmer, Waltham).

### Statistical Analysis

Data regarding cytokine levels and gene expression were obtained from six independent experiments, using cells derived from a different donor in each experiment, and with duplicate wells for each condition. The mean value of these duplicate measurements was used for further analysis. Epithelial permeability was assessed in three independent experiments. Data on degradation of recombinant IL-6 and test of inhibitors were generated in three to four independent experiments. Cytokine concentrations and fold changes were logarithmically transformed to approximately conform to normality. For analysis, values below the minimum quantifiable level (MQL) were used as 0.5*MQL value of the respective assay. Statistical significance was tested by analysis of variance (ANOVA) and Tukey’s multiple comparison test for comparison between all experimental groups, or Dunnett’s multiple comparison test for comparison of densitometry data (**Figures 3C, D**, comparison to input), all with a significance level of α = 0.05. Figures and text show untransformed values (mean +/- SD) to facilitate interpretation.

## Results

### Epithelial Cytokine Secretion in Response to HRV Is Altered in Cultures Pre-Infected With *P. aeruginosa*

We first sought to assess the impact of chronic bacterial infection on the production of inflammatory mediators during secondary viral infection. Therefore, protein concentrations and mRNA levels of key cytokines and chemokines were measured in the basal medium and cell lysates of air-liquid interface cultures following chronic PA infection and acute HRV superinfection.

Concentrations of IL-1β in basolateral media were on average below 10 pg/ml (MQL) for sham-treated cells and for cells exposed to either pathogen alone, but were significantly higher in cells that had been infected with HRV16 following a prior exposure to the non-mucoid PA isolate (28.88 ± 17.69 pg/ml vs. 6.10 ± 2.69 pg/ml, p< 0.001, **Figure 1B**). Likewise, pro-IL-1β mRNA levels were significantly higher in cells co-infected with the non-mucoid PA and HRV16 than in cells infected with HRV16 alone (1019 ± 1431 fold change vs. 28 ± 35 fold change relative to uninfected controls, p=0.012, **Figure 1C**). PA infection alone had no significant effect on concentrations of IL-6, whereas IL-6 production was strongly induced by infection with HRV16 (2301 ± 1873 pg/ml, **Figure 1D**). Strikingly, in cells co-infected with non-mucoid PA and HRV16, virus-induced elevation of IL-6 was almost entirely abrogated (53.17 ± 58.84 pg/ml, p< 0.001 vs. PA-/HRV+), whereas IL-6 concentrations in media of cells co-infected with HRV16 and a mucoid PA isolate were similar to those of cells infected with the virus alone (1876 ± 1067 pg/ml). On mRNA level, compared to HRV infection alone (96 ± 127 fold change), co-infection with the non-mucoid PA isolate significantly increased IL-6 mRNA (4742 ± 8020 fold change, p = 0.04), whereas co-infection with the mucoid isolate did not (79 ± 108, p = 0.999, **Figure 1E**).

**Figure 2:**
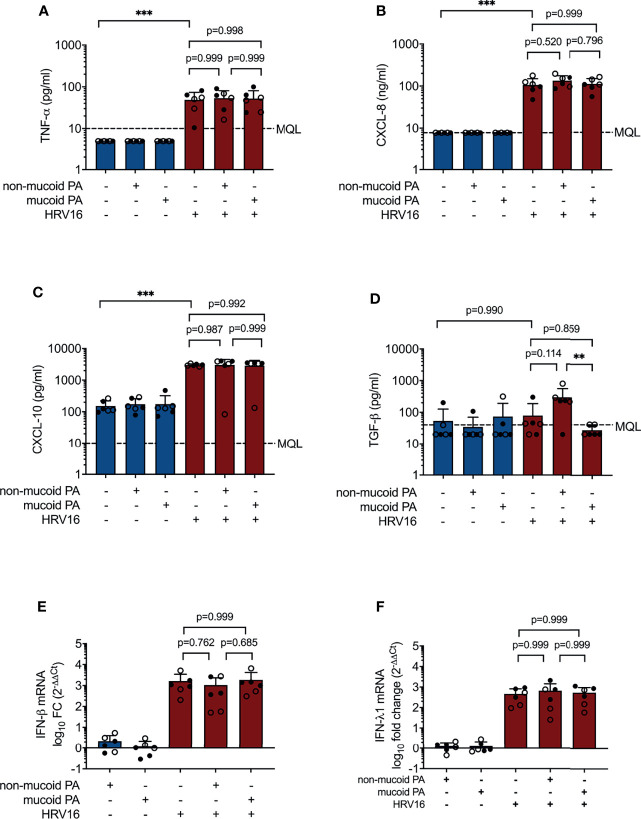
Cytokine production in differentiated bronchial epithelial cells co-infected with *P. aeruginosa* (PA) and human rhinovirus 16 (HRV16). **(A)** TNFα protein concentrations in basal media **(B)** CXCL-8 protein concentrations in basal media **(C)** CXCL-10 protein concentrations in basal media **(D)** TGF-β protein concentrations in basal media **(E)** IFN-β mRNA levels **(F)** IFN- λ mRNA levels. MQL, Minimum quantifiable level. Cell type: ○ Emphysema ● Cystic fibrosis. Data were obtained in six independent experiments, each performed with cells derived from a different donor. *p ≤ 0.05, **p ≤ 0.01, ***p ≤ 0.001.

Other cytokines and chemokines, including CXCL-8 (IL-8), CXCL-10 (IP-10), TGF-β and TNF-α protein, and type I IFN (IFN-β) and type III IFN (IFN-λ1) mRNA were increased to a similar extent during viral infection and co-infections with either of the PA isolates and HRV16. However, TGF-β was significantly increased in co-infection with the non-mucoid PA isolate compared to co-infection with the mucoid PA isolate (298 ± 264 pg/ml vs. 27± 11 pg/ml, p= 0.008, **Figure 2**). Protein and mRNA levels of the molecules listed above were similar in cells isolated from lung explants emphysema patients and those from CF patients.

### Proteolytic Degradation of IL-6 During Co-Infection of Bronchial Epithelial Cells With *P. aeruginosa* and HRV16

**Figure 3:**
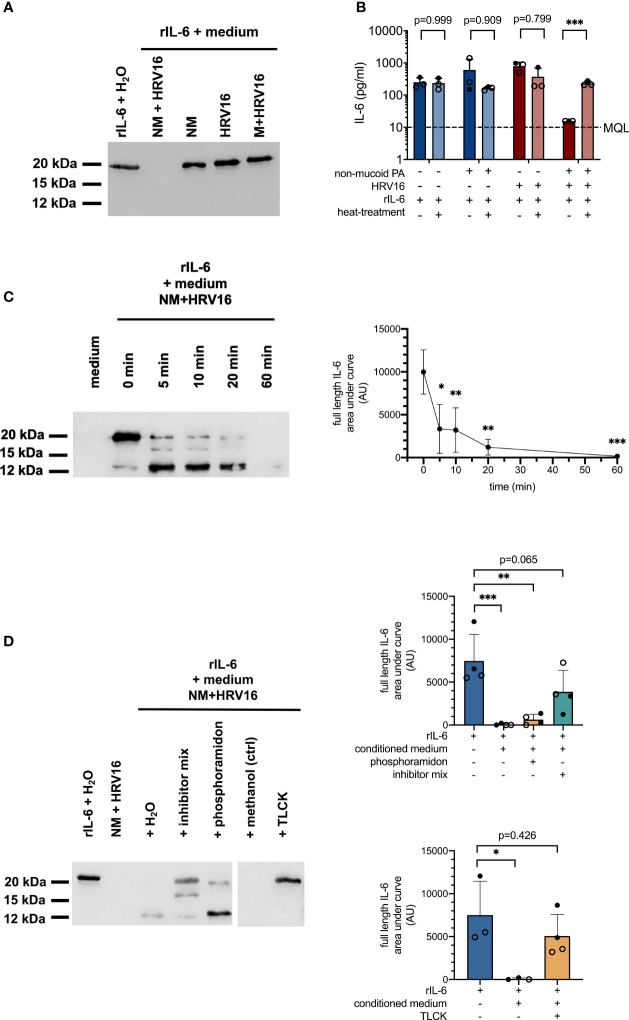
Proteolytic degradation of recombinant IL-6 by conditioned media from PA/HRV16 co-infections. **(A)** Western Blot (IL-6) for rIL-6 incubated with conditioned media or H_2_O for 4 h (IL-6). **(B)** ELISA (IL-6) of rIL-6 incubated with either untreated or heat-inactivated (95°C/5 min) conditioned media obtained from infection experiments. **(C)** Time course series: rIL-6 was incubated with conditioned media obtained from non-mucoid PA+HRV16 co-infections for the indicated times and samples were subsequently analyzed by Western Blot and densitometry for full length IL-6. **(D)** rIL-6 was incubated with conditioned media in the presence of the indicated protease inhibitors and samples were analyzed by Western Blot and densitometry for full length IL-6. NM, non-mucoid PA isolate; M, mucoid PA isolate. Cell type: ○ Emphysema ● Cystic fibrosis. Western blots **(A, C, D)** are representative images of three to four independent experiments. ELISA data **(B)** and densitometry show mean +/- SD of three independent experiments. *p ≤ 0.05, **p ≤ 0.01, ***p ≤ 0.001.

A significant increase in IL-6 mRNA levels in conjunction with absence of IL-6 protein suggested either degradation or post-transcriptional regulation of IL-6 production in cells co-infected with non-mucoid PA and HRV16. In order to elucidate the divergent effects of PA/HRV co-infection on IL-6 mRNA and protein levels in bronchial epithelial cells, we tested if cell-free conditioned basal media of co-infected cells contain soluble factors that are able to degrade recombinant IL-6 (rIL-6). To this end, media from different infection conditions were mixed with 1 µg rIL-6 and incubated for 4 h at 37°C. Western blot analysis showed that rIL-6 was entirely degraded when mixed with conditioned media obtained from cells co-infected with a non-mucoid PA isolate and HRV16 (**Figure 3A**). In contrast, rIL-6 was not affected by conditioned media of any of the other tested infection conditions, including infection with the individual pathogens, and media from a co-infection with a mucoid PA isolate and HRV16. Similar results were obtained when rIL-6 was mixed with apical wash fluid of the infected cultures instead of culture medium (data not shown).

In line with the results obtained by Western Blot, rIL-6 was no longer detectable by ELISA in samples incubated with media from cells co-infected with non-mucoid PA and HRV16 (**Figure 3B**). If media were heat-inactivated prior to mixing with the recombinant protein, concentrations of IL-6 were comparable to those of controls, suggesting degradation of IL-6 by a heat-labile mediator. A time course series showed two distinct major cleavage products of rIL-6 occurring within minutes after exposure to conditioned media of co-infected cells, with a molecular weight of approximately 16 and 12 kDa respectively (full length IL-6: 21 kDa). Densitometric analysis showed a statistically significant reduction in full length IL-6 already after five minutes. (**Figure 3C**, right panel). Addition of a protease inhibitor cocktail (cOmplete EDTA-free Protease Inhibitor Cocktail, Roche) was able to partially inhibit proteolytic degradation of full length rIL-6, while the metalloprotease inhibitor phosphoramidon only inhibited degradation of the 12 kDa fragment. In contrast, addition of the protease inhibitor TLCK, predominantly inhibiting serine proteases, was able to completely block proteolytic degradation of rIL-6 at a final concentration of 40 mM (**Figure 3D**).

### PA/HRV Co-Infection Compromises Epithelial Barrier

**Figure 4:**
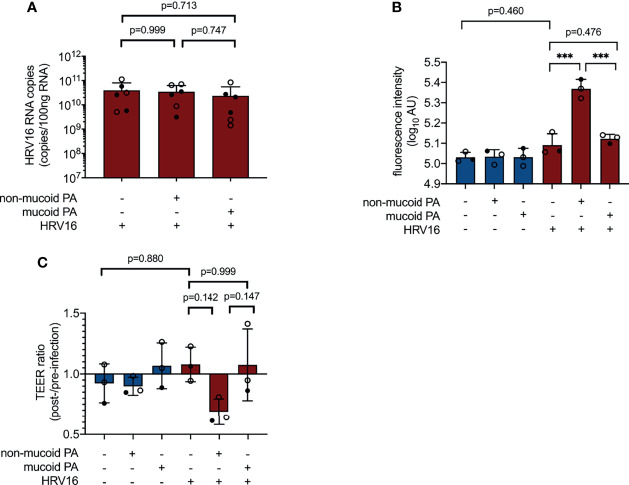
Impact of PA/HRV co-infection on viral load and epithelial barrier function. **(A)** Viral RNA load in cell lysates 48 h post infection (RT qPCR) **(B)** Permeability of cell layers to FITC-labeled dextran 48 h after (co-) infection **(C)** Ratio of transepithelial electrical resistance (TEER) prior to HRV infection and 48 h after HRV infection. AU, arbitrary unit, cell type:○ Emphysema ● Cystic fibrosis. Data were obtained in six **(A)** and three **(B, C)** independent experiments, each performed with cells derived from a different donor. *p ≤ 0.05, **p ≤ 0.01, ***p ≤ 0.001.

Co-infection with the non-mucoid PA isolate and HRV16 appeared to cause more pronounced damage to the cell layer than any of the other infection conditions tested, however, this was not associated with an increase in viral load (**Figure 4A**). As a measure for permeability of the epithelial layer, we measured leakage of FITC-labeled dextran (average molecular weight 20 kDa) from the apical compartment of the transwell system into the basal chamber. Fluorescence intensity in samples taken from the basal compartment of cultures co-infected with the non-mucoid PA isolate and HRV16 was significantly higher than in sham-infected controls or cells infected with HRV16 only (**Figure 4B**). This was not the case for any of the other infection conditions.

As a second method to assess barrier integrity in infected cultures, we determined transepithelial electrical resistance (TEER). Differences between groups failed to reach statistical significance at 48 h post viral infection (NM+/HRV+ vs. PA-/HRV+, p=0.142, **Figure 4C**), but showed a similar overall trend as FITC dextran leakage assay.

### Altered Expression of Differentiation-Associated Genes During PA/HRV Co-Infection

**Figure 5:**
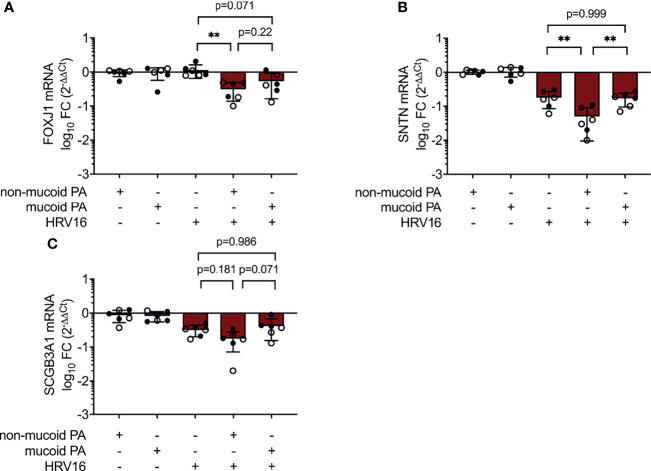
Altered expression of differentiation-associated genes during PA/HRV co-infection. mRNA levels were assessed in cell lysates 48h post infection by RT qPCR. **(A)** Forkhead Box J1(FOXJ1) **(B)** Sentan, Cilia Apical Structure Protein (SNTN) **(C)** Secretoglobin Family 3A Member 1 (SCGB3A1). FC: fold change. Cell type, ○ Emphysema ● Cystic fibrosis. Data were obtained in six independent experiments, each performed with cells derived from a different donor. *p ≤ 0.05, **p ≤ 0.01, ***p ≤ 0.001.

IL-6 is a key player in regulation of epithelial repair after virus-induced lung injury (Yang et al., 2017). Ciliated cell differentiation of basal cells is dependent on the transcriptional regulator Forkhead box J1 (FOXJ1), which can be activated through the IL-6/STAT3 pathway (Tadokoro et al., 2014). We found FOXJ1 mRNA levels to be decreased in co-infection with the non-mucoid PA isolate, compared to virus-infection alone (0.31 ± 0.17 fold change vs. 1.14 ± 0.49 fold change, p=0.002) (**Figure 5A**). This was accompanied by a statistical significant decrease in the expression of Cilia Apical Structure Protein (SNTN) in cells co-infected with the non-mucoid PA isolate and HRV16 (0.05 ± 0.04 fold change vs. 0.18 ± 0.09 fold change, p=0.002) (**Figure 5B**). On the other hand, the differences in mRNA levels of Secretoglobin (SCGB3A1), a product of secretory cells, did not reach statistical significance between cells co-infected with the non-mucoid PA isolate and single HRV16 infection (0.18 ± 0.11 fold change vs. 0.32 ± 0.12 fold change, p=0.188) (**Figure 5C**).

## Discussion

In this study, we show that non-mucoid PA can modulate innate antiviral responses of respiratory epithelial cells on several levels, most strikingly by an almost complete protease-mediated degradation of virus-induced IL-6. Moreover, co-infection with non-mucoid PA and HRV16 affected epithelial barrier integrity and led to differences in the expression of genes involved in epithelial cell differentiation in our model.

In this *in vitro* model, we observed pronounced differences between an isogenic non-mucoid and a mucoid PA isolate recovered from the same respiratory sample of one CF patient. While co-infection with the non-mucoid isolate and HRV16 led to almost complete degradation of endogenous and exogenous IL-6, co-infections with the mucoid isolate did not, which might, in part, be explained by altered expression, regulation or secretion of bacterial proteases.

In CF, PA adapts genetically during chronic infection and is frequently found to be less virulent compared with environmental isolates and those recovered from early CF lung infection (Folkesson et al., 2012). Adaptive mutations can result in the switch to an alginate-overproducing, mucoid phenotype, but also in the loss of function or downregulation of various virulence factors. Mucoid PA can revert to the non-mucoid phenotype in chronic infections, and both can regularly be isolated simultaneously (Hogardt and Heesemann, 2010). PA possesses several proteases, including elastase B (LasB) and alkaline protease (AprA) which are known to degrade human cytokines, including IL-6 (LaFayette et al., 2015; Saint-Criq et al., 2018; Sorensen et al., 2020). Both are regulated by the quorum sensing transcription factor LasR, which is prone to loss of function mutations in chronic infection (Smith et al., 2006; Hoffman et al., 2009). Even though the exact protease mediating the effects we observed in our model remains to be identified, alterations in the PA genome are likely to contribute to the isolate-specific effects we observed. Inhibition of protease activity by the inhibitor TLCK moreover suggests that degradation of IL-6 is mediated by a serine protease. Interestingly, co-infection with HRV16 was required to induce the degradation of IL-6 in our model, and bacterial infection alone had no effect on IL-6 levels. These findings indicate that next to genetic differences, IL-6 degradation might also be affected by viral infection of the host cell.

PA proteases have previously been described to also target CXCL-8 (LaFayette et al., 2015; Saint-Criq et al., 2018) and IFN-λ (Sorensen et al., 2020). In our model, we found the effects of co-infection with PA and HRV16 on CXCL-8 to be similar to those of HRV infection alone. Moreover, viral load was comparable between all conditions with virus-infections, suggesting no relevant impairment of mediators conferring an antiviral state (such as type I/III IFNs) in epithelial cells.

Individual infections alone, including HRV, did not significantly induce IL-1β release in our model. HRV has previously been described to increase release of IL-1β in undifferentiated airway epithelial cells (Terajima et al., 1997; Piper et al., 2013; Ling et al., 2020). However, in line with our studies, other groups using air liquid interface cultures more similar to ours likewise did not observe an increase in pro-IL-1β mRNA (Lopez-Souza et al., 2009) or IL-1β protein (Hill et al., 2016). In contrast to viral infection alone, we detected a moderate, yet statistically significant increase in pro-IL-1β mRNA and IL-1β protein levels in cells co-infected with the non-mucoid PA isolate and HRV. IL-1β plays a key role in induction of pro-inflammatory signals, recruitment and activation of immune cells (Piper et al., 2013). Of note, IL-1ß can also upregulate gene expression of MUC5AC and MUC5B, leading to higher production of these glycoproteins and higher amount of mucus – a typical symptom in CF exacerbations (Fujisawa et al., 2011; Chen et al., 2014). In pBECs of pediatric CF patients, the level of HRV-induced IL-1ß moreover was found to correlate with the amount of necrotic cell death (Montgomery et al., 2018). Co-infections with certain PA strains and respiratory viruses might therefore aggravate pulmonary inflammation and tissue damage in CF, while impairing other important innate immune mechanisms.

The interaction between PA and HRV could be mediated by changes in the microenvironment during infection, which can impact on bacterial growth, lifestyle and virulence. Virus-induced changes that can influence bacterial growth patterns include increased oxidative stress, but also alterations in the bioavailability of nutrients and growth factors for bacteria (Chattoraj et al., 2011a; Siegel et al., 2014; Hendricks et al., 2016; Hendricks et al., 2021). In our model, the effects of co-infection with HRV were dependent on the PA isolate, indicating the differences are not exclusively attributable to altered environmental conditions. However, differences in metabolism or growth characteristics of the two isolates could have influenced their reaction towards an altered microenvironment during co-infection with HRV. Likewise, these conditions could contribute to an increased expression of bacterial proteases during co-infections. Alternatively, direct interactions between the bacteria and HRV virions might contribute to altered virulence. Such interactions have previously been described to occur between *S. pneumoniae* and Respiratory Syncytial Virus (RSV) through binding of the *S. pneumoniae* penicillin binding protein 1a to the RSV G protein (Smith et al., 2014). This binding enhances the expression of key bacterial virulence factors and strongly increases pneumococcal virulence in a mouse model. This particular mechanism appears to be very pathogen specific, and it is thus far unclear if similar interactions are relevant for other combinations of viral and bacterial pathogens as well.

Next to modulation of cytokine expression, we found that co-infection with the non-mucoid PA isolate and HRV also negatively affected epithelial barrier integrity. In acute infection models, both, PA and rhinoviruses, have previously been shown to be able to individually compromise epithelial barrier function (Sajjan et al., 2008; Looi et al., 2018; Li et al., 2019; Michi et al., 2021). Strikingly, in our model of chronic PA infection using a very low bacterial load over a prolonged period, we only observed a marked increase in epithelial permeability in cells co-infected with a non-mucoid PA isolate and HRV, whereas individual infections or co-infections with a mucoid PA isolate and HRV had no effect on permeability of the epithelial layer to FITC-labeled dextran. The discrepant results between our data and previously published findings regarding HRV-induced disruption of the epithelial barrier are likely to be due to methodological differences between the studies. These include the time after infection when the measurements were conducted, the viral strains used (Michi et al., 2021), and the molecular weight of the FITC-labeled dextran used to assess permeability (Looi et al., 2018).

The increased damage to the epithelial cell layer could also be a result of an increased virulence of the non-mucoid PA isolate. Additionally, IL-6 plays a role in epithelial repair following lung injury (Tadokoro et al., 2014; Saint-Criq et al., 2018), hence the proteolytic degradation of this cytokine during non-mucoid PA/HRV infection might hamper the repair of pathogen-induced damage. In line with this, we found mRNA levels of markers of epithelial cell differentiation to ciliated cells (transcription factor FOXJ1 and the ciliary apical structure protein SNTN) to be lower following co-infection with non-mucoid PA and HRV than after viral infection alone. This finding could indicate that co-infection with non-mucoid PA and HRV affects epithelial cell type distribution and/or repair mechanisms, possibly due to lack of IL-6 under these conditions.

To date, there is only little clinical data on the interplay of colonizing bacteria and viral infections in CF. Previous studies do not indicate a generalizable increased susceptibility to acquire a respiratory viral infection in CF patients infected with PA (Flight et al., 2014; Esther et al., 2014; Meyer et al., 2020). However, a recent study found that a certain subgroup of CF patients, i.e. those intermittently infected with PA, more frequently tests positive for HRV in samples from the lower respiratory tract and moreover exhibits on average higher viral loads (Sorensen et al., 2020), which at least in part, might be related to the expression of virulence factors by PA.

We observed clear differences in the modulation of virus infection between two phenotypical distinguishable, isogenic PA isolates, both obtained from a CF patient chronically infected with PA. Therefore, we suggest that differences in viral infection depend on phenotypical differences and differences in virulence factors rather than on the duration of PA infection alone.

Of note, we did not observe significant differences in cytokine levels between cells isolated from lung tissue of CF patients and those isolated from tissue of lung emphysema patients. Potentially, differences between cell types were not pronounced enough compared to differences between infection conditions. Although our findings regarding the inflammatory response appear to be independent of CFTR malfunction or deficiency, they are particularly relevant in adults with CF, the majority of whom are chronically infected with PA. Nevertheless, chronic pulmonary PA infections are also observed in other diseases of the respiratory tract, like non-CF bronchiectasis (Woo et al., 2018) or chronic obstructive pulmonary disease (COPD) (Martinez-Garcia et al., 2021), and might therefore also affect the course of viral infections in these settings.

To date, mechanisms underlying bacterial-viral interactions have largely been studied *in vitro* using acute infection models, with relatively high bacterial loads and/or short infection times (Sajjan et al., 2006; Van Ewijk et al., 2007; Chattoraj et al., 2011b; Bellinghausen et al., 2016). Even though these studies have revealed several processes relevant to co-infections, they are limited as they cannot consider effects that arise due to adaptation or tolerance induced over time on the one hand, and accumulation of bacterial products on the other hand. By using a model of differentiated primary bronchial epithelial cells repeatedly exposed to clinical PA isolates, we could consider these factors and establish a model for studying interactions of PA and respiratory viruses in the context of chronic bacterial infection. Even though this model does currently not depict the influence of immune cells, macrophages or neutrophils could be integrated in such cultures in the future. Similarly, co-culture models of epithelial cells and fibroblasts, as have been established in other settings (Ishikawa et al., 2017), could provide useful insights into repair mechanisms following co-infections.

In summary, our experimental data show that the interaction between chronic PA and acute viral infection can profoundly alter the epithelial response to these pathogens. These changes encompass a modulation of cytokine production, proteolytic degradation of IL-6 and a compromised epithelial barrier function. Interactions between bacterial and viral infections, and also the role of the microbiota in shaping susceptibility and response to viral infections, are still insufficiently understood. A better understanding of these host-pathogen and pathogen-pathogen interactions could help to improve risk assessments and management for individuals with chronic pulmonary PA infections.

## Data Availability Statement

The original contributions presented in the study are included in the article/**Supplementary Material**. Further inquiries can be directed to the corresponding author.

## Author Contributions

AE, CH, GR, and CB conceptualized the project and designed the experiments. AE, HB, MH, RS, DJ, PB, and CB performed the experiments and acquisition of data and material. AE and CB analyzed the data and wrote the manuscript. CH, GR, MH, DJ, PB, and CB acquired funding and provided resources. All authors revised the manuscript and agreed to its submission.

## Funding

The work presented in this manuscript was partially funded by Gilead Sciences, Grant Program Infectious Diseases, Germany. The funders had no role in study design, data collection and analysis, decision to publish or preparation of the manuscript.

## Conflict of Interest

The work presented in this manuscript was partially funded by Gilead Sciences, Grant Program Infectious Diseases, Germany. The funders had no role in study design, data collection and analysis, decision to publish or preparation of the manuscript.

## Publisher’s Note

All claims expressed in this article are solely those of the authors and do not necessarily represent those of their affiliated organizations, or those of the publisher, the editors and the reviewers. Any product that may be evaluated in this article, or claim that may be made by its manufacturer, is not guaranteed or endorsed by the publisher.

## References

[B1] Bellinghausen C. Gulraiz F. Heinzmann A. C. Dentener M. A. Savelkoul P. H. Wouters E. F.. (2016). Exposure to Common Respiratory Bacteria Alters the Airway Epithelial Response to Subsequent Viral Infection. Respir. Res. 17 (1), 68. doi: 10.1186/s12931-016-0382-z 27259950 PMC4891894

[B2] Bergeron C. Cantin A. M. (2019). Cystic Fibrosis: Pathophysiology of Lung Disease. Semin. Respir. Crit. Care Med. 40 (6), 715–726. doi: 10.1055/s-0039-1694021 31659725

[B3] Chattoraj S. S. Ganesan S. Faris A. Comstock A. Lee W. M. Sajjan U. S. (2011b). Pseudomonas Aeruginosa Suppresses Interferon Response to Rhinovirus Infection in Cystic Fibrosis But Not in Normal Bronchial Epithelial Cells. Infect. Immun. 79 (10), 4131–4145. doi: 10.1128/IAI.05120-11 21825067 PMC3187241

[B4] Chattoraj S. S. Ganesan S. Jones A. M. Helm J. M. Comstock A. T. Bright-Thomas R.. (2011a). Rhinovirus Infection Liberates Planktonic Bacteria From Biofilm and Increases Chemokine Responses in Cystic Fibrosis Airway Epithelial Cells. Thorax 66 (4), 333–339. doi: 10.1136/thx.2010.151431 21289024 PMC3208236

[B5] Chen Y. Garvin L. M. Nickola T. J. Watson A. M. Colberg-Poley A. M. Rose M. C. (2014). IL-1beta Induction of MUC5AC Gene Expression is Mediated by CREB and NF-kappaB and Repressed by Dexamethasone. Am. J. Physiol. Lung Cell Mol. Physiol. 306 (8), L797–L807. doi: 10.1152/ajplung.00347.2013 24487386 PMC3989721

[B6] de Steenhuijsen Piters W. A. Heinonen S. Hasrat R. Bunsow E. Smith B. Suarez-Arrabal M. C.. (2016). Nasopharyngeal Microbiota, Host Transcriptome, and Disease Severity in Children With Respiratory Syncytial Virus Infection. Am. J. Respir. Crit. Care Med. 194 (9), 1104–1115. doi: 10.1164/rccm.201602-0220OC 27135599 PMC5114450

[B7] Ederveen T. H. A. Ferwerda G. Ahout I. M. Vissers M. de Groot R. Boekhorst J.. (2018). Haemophilus is Overrepresented in the Nasopharynx of Infants Hospitalized With RSV Infection and Associated With Increased Viral Load and Enhanced Mucosal CXCL8 Responses. Microbiome 6 (1), 10. doi: 10.1186/s40168-017-0395-y 29325581 PMC5765694

[B8] Esther C. R. Lin F. C. Kerr A. Miller M. B. Gilligan P. H. (2014). Respiratory Viruses are Associated With Common Respiratory Pathogens in Cystic Fibrosis. Pediatr. Pulmonol. 49 (9), 926–931. doi: 10.1002/ppul.22917 24167159

[B9] Flight W. G. Bright-Thomas R. J. Tilston P. Mutton K. J. Guiver M. Morris J.. (2014). Incidence and Clinical Impact of Respiratory Viruses in Adults With Cystic Fibrosis. Thorax 69 (3), 247–253. doi: 10.1136/thoraxjnl-2013-204000 24127019

[B10] Folkesson A. Jelsbak L. Yang L. Johansen H. K. Ciofu O. Høiby N.. (2012). Adaptation of Pseudomonas Aeruginosa to the Cystic Fibrosis Airway: An Evolutionary Perspective. Nat. Rev. Microbiol. 10 (12), 841–851. doi: 10.1038/nrmicro2907 23147702

[B11] Fujisawa T. Chang M. M. Velichko S. Thai P. Hung L. Y. Huang F.. (2011). NF-kappaB Mediates IL-1beta- and IL-17A-Induced MUC5B Expression in Airway Epithelial Cells. Am. J. Respir. Cell Mol. Biol. 45 (2), 246–252. doi: 10.1165/rcmb.2009-0313OC 20935193 PMC3175554

[B12] Garcia-Clemente M. de la Rosa D. Maiz L. Giron R. Blanco M. Olveira C.. (2020). Impact of Pseudomonas Aeruginosa Infection on Patients With Chronic Inflammatory Airway Diseases. J. Clin. Med. 9 (12), 3800. doi: 10.3390/jcm9123800 PMC7760986 33255354

[B13] Gulraiz F. Bellinghausen C. Bruggeman C. A. Stassen F. R. (2015). Haemophilus Influenzae Increases the Susceptibility and Inflammatory Response of Airway Epithelial Cells to Viral Infections. FASEB J. 29 (3), 849–858. doi: 10.1096/fj.14-254359 25411435

[B14] Hendricks M. R. Lane S. Melvin J. A. Ouyang Y. Stolz D. B. Williams J. V.. (2021). Extracellular Vesicles Promote Transkingdom Nutrient Transfer During Viral-Bacterial Co-Infection. Cell Rep. 34 (4), 108672. doi: 10.1016/j.celrep.2020.108672 33503419 PMC7918795

[B15] Hendricks M. R. Lashua L. P. Fischer D. K. Flitter B. A. Eichinger K. M. Durbin J. E.. (2016). Respiratory Syncytial Virus Infection Enhances Pseudomonas Aeruginosa Biofilm Growth Through Dysregulation of Nutritional Immunity. Proc. Natl. Acad. Sci. U. S. A. 113 (6), 1642–1647. doi: 10.1073/pnas.1516979113 26729873 PMC4760822

[B16] Hiemstra P. S. McCray P. B. Bals R. (2015). The Innate Immune Function of Airway Epithelial Cells in Inflammatory Lung Disease. Eur. Respir. J. 45 (4), 1150–1162. doi: 10.1183/09031936.00141514 25700381 PMC4719567

[B17] Hierholzer J. C. Killington R. A. (1996). “Virus Isolation and Quantitation,” in Virology Methods Manual. Eds. Mahy B. W. J. Kangro H. O. (London: Academic Press Ltd), 36–37.

[B18] Hill A. R. Donaldson J. E. Blume C. Smithers N. Tezera L. Tariq K.. (2016). IL-1α Mediates Cellular Cross-Talk in the Airway Epithelial Mesenchymal Trophic Unit. Tissue Barriers 4 (3), e1206378. doi: 10.1080/21688370.2016.1206378 27583193 PMC4993579

[B19] Hoffman L. R. Kulasekara H. D. Emerson J. Houston L. S. Burns J. L. Ramsey B. W.. (2009). Pseudomonas Aeruginosa lasR Mutants are Associated With Cystic Fibrosis Lung Disease Progression. J. Cyst Fibros 8 (1), 66–70. doi: 10.1016/j.jcf.2008.09.006 18974024 PMC2631641

[B20] Hogardt M. Heesemann J. (2010). Adaptation of Pseudomonas Aeruginosa During Persistence in the Cystic Fibrosis Lung. Int. J. Med. Microbiol. 300 (8), 557–562. doi: 10.1016/j.ijmm.2010.08.008 20943439

[B21] Ichinohe T. Pang I. K. Kumamoto Y. Peaper D. R. Ho J. H. Murray T. S.. (2011). Microbiota Regulates Immune Defense Against Respiratory Tract Influenza A Virus Infection. Proc. Natl. Acad. Sci. U. S. A. 108 (13), 5354–5359. doi: 10.1073/pnas.1019378108 21402903 PMC3069176

[B22] Ishikawa S. Ishimori K. Ito S. (2017). A 3D Epithelial-Mesenchymal Co-Culture Model of Human Bronchial Tissue Recapitulates Multiple Features of Airway Tissue Remodeling by TGF-Beta1 Treatment. Respir. Res. 18 (1), 195. doi: 10.1186/s12931-017-0680-0 29166920 PMC5700468

[B23] LaFayette S. L. Houle D. Beaudoin T. Wojewodka G. Radzioch D. Hoffman L. R.. (2015). Cystic Fibrosis-Adapted. Sci. Adv. 1 (6), e1500199. doi: 10.1126/sciadv.1500199 26457326 PMC4597794

[B24] Ling K. M. Garratt L. W. Gill E. E. Lee A. H. Y. Agudelo-Romero P. Sutanto E. N.. (2020). Rhinovirus Infection Drives Complex Host Airway Molecular Responses in Children With Cystic Fibrosis. Front. Immunol. 11. doi: 10.3389/fimmu.2020.01327 PMC7378398 32765492

[B25] Li J. Ramezanpour M. Fong S. A. Cooksley C. Murphy J. Suzuki M.. (2019). Pseudomonas Aeruginosa Exoprotein-Induced Barrier Disruption Correlates With Elastase Activity and Marks Chronic Rhinosinusitis Severity. Front. Cell Infect. Microbiol. 9. doi: 10.3389/fcimb.2019.00038 PMC6400838 30873390

[B26] Livak K. J. Schmittgen T. D. (2001). Analysis of Relative Gene Expression Data Using Real-Time Quantitative PCR and the 2(-Delta Delta C(T)) Method. Methods 25 (4), 402–408. doi: 10.1006/meth.2001.1262 11846609

[B27] Looi K. Buckley A. G. Rigby P. J. Garratt L. W. Iosifidis T. Zosky G. R.. (2018). Effects of Human Rhinovirus on Epithelial Barrier Integrity and Function in Children With Asthma. Clin. Exp. Allergy 48 (5), 513–524. doi: 10.1111/cea.13097 29350877

[B28] Lopez-Souza N. Favoreto S. Wong H. Ward T. Yagi S. Schnurr D.. (2009). *In Vitro* Susceptibility to Rhinovirus Infection is Greater for Bronchial Than for Nasal Airway Epithelial Cells in Human Subjects. J. Allergy Clin. Immunol. 123 (6), 1384–1390.e2. doi: 10.1016/j.jaci.2009.03.010 19428098 PMC2744461

[B29] Malhotra S. Hayes D. Wozniak D. J. (2019). Cystic Fibrosis and Pseudomonas Aeruginosa: The Host-Microbe Interface. Clin. Microbiol. Rev. 32 (3), e00138–18. doi: 10.1128/CMR.00138-18 PMC6589863 31142499

[B30] Martinez-Garcia M. Faner R. Oscullo G. la Rosa-Carrillo D. Soler-Cataluña J. J. Ballester M.. (2021). Chronic Bronchial Infection and Incident Cardiovascular Events in Chronic Obstructive Pulmonary Disease Patients: A Long-Term Observational Study. Respirology 26 (8), 776–785. doi: 10.1111/resp.14086 34002922

[B31] Meyer V. M. C. Siqueira M. M. Costa P. F. B. M. Caetano B. C. Oliveira Lopes J. C. Folescu T. W.. (2020). Clinical Impact of Respiratory Virus in Pulmonary Exacerbations of Children With Cystic Fibrosis. PLoS One 15 (10), e0240452. doi: 10.1371/journal.pone.0240452 33112873 PMC7592759

[B32] Michi A. N. Yipp B. G. Dufour A. Lopes F. Proud D. (2021). PGC-1α Mediates a Metabolic Host Defense Response in Human Airway Epithelium During Rhinovirus Infections. Nat. Commun. 12 (1), 3669. doi: 10.1038/s41467-021-23925-z 34135327 PMC8209127

[B33] Montgomery S. T. Dittrich A. S. Garratt L. W. Turkovic L. Frey D. L. Stick S. M.. (2018). Interleukin-1 is Associated With Inflammation and Structural Lung Disease in Young Children With Cystic Fibrosis. J. Cyst Fibros 17 (6), 715–722. doi: 10.1016/j.jcf.2018.05.006 29884450

[B34] Piper S. C. Ferguson J. Kay L. Parker L. C. Sabroe I. Sleeman M. A.. (2013). The Role of Interleukin-1 and Interleukin-18 in Pro-Inflammatory and Anti-Viral Responses to Rhinovirus in Primary Bronchial Epithelial Cells. PLoS One 8 (5), e63365. doi: 10.1371/journal.pone.0063365 23723976 PMC3665753

[B35] Saint-Criq V. Villeret B. Bastaert F. Kheir S. Hatton A. Cazes A.. (2018). Pseudomonas Aeruginosa LasB Protease Impairs Innate Immunity in Mice and Humans by Targeting a Lung Epithelial Cystic Fibrosis Transmembrane Regulator-IL-6-Antimicrobial-Repair Pathway. Thorax 73 (1), 49–61. doi: 10.1136/thoraxjnl-2017-210298 28790180 PMC5738602

[B36] Sajjan U. S. Jia Y. Newcomb D. C. Bentley J. K. Lukacs N. W. LiPuma J. J.. (2006). H. Influenzae Potentiates Airway Epithelial Cell Responses to Rhinovirus by Increasing ICAM-1 and TLR3 Expression. FASEB J. 20 (12), 2121–2123. doi: 10.1096/fj.06-5806fje 16914605

[B37] Sajjan U. Wang Q. Zhao Y. Gruenert D. C. Hershenson M. B. (2008). Rhinovirus Disrupts the Barrier Function of Polarized Airway Epithelial Cells. Am. J. Respir. Crit. Care Med. 178 (12), 1271–1281. doi: 10.1164/rccm.200801-136OC 18787220 PMC2599868

[B38] Siegel S. J. Roche A. M. Weiser J. N. (2014). Influenza Promotes Pneumococcal Growth During Coinfection by Providing Host Sialylated Substrates as a Nutrient Source. Cell Host Microbe 16 (1), 55–67. doi: 10.1016/j.chom.2014.06.005 25011108 PMC4096718

[B39] Smith E. E. Buckley D. G. Wu Z. Saenphimmachak C. Hoffman L. R. D’Argenio D. A.. (2006). Genetic Adaptation by Pseudomonas Aeruginosa to the Airways of Cystic Fibrosis Patients. Proc. Natl. Acad. Sci. U. S. A. 103 (22), 8487–8492. doi: 10.1073/pnas.0602138103 16687478 PMC1482519

[B40] Smith C. M. Sandrini S. Datta S. Freestone P. Shafeeq S. Radhakrishnan P.. (2014). Respiratory Syncytial Virus Increases the Virulence of Streptococcus Pneumoniae by Binding to Penicillin Binding Protein 1a. A New Paradigm in Respiratory Infection. Am. J. Respir. Crit. Care Med. 190 (2), 196–207. doi: 10.1164/rccm.201311-2110OC 24941423 PMC4226051

[B41] Sonawane A. R. Tian L. Chu C. Y. Qiu X. Wang L. Holden-Wiltse J.. (2019). Microbiome-Transcriptome Interactions Related to Severity of Respiratory Syncytial Virus Infection. Sci. Rep. 9 (1), 13824. doi: 10.1038/s41598-019-50217-w 31554845 PMC6761288

[B42] Sorensen M. Kantorek J. Byrnes L. Boutin S. Mall M. A. Lasitschka F.. (2020). Pseudomonas Aeruginosa Modulates the Antiviral Response of Bronchial Epithelial Cells. Front. Immunol. 11. doi: 10.3389/fimmu.2020.00096 PMC7025480 32117250

[B43] Tadokoro T. Wang Y. Barak L. S. Bai Y. Randell S. H. Hogan B. L. (2014). IL-6/STAT3 Promotes Regeneration of Airway Ciliated Cells From Basal Stem Cells. Proc. Natl. Acad. Sci. U.S. A. 111 (35), E3641–E3649. doi: 10.1073/pnas.1409781111 25136113 PMC4156689

[B44] Terajima M. Yamaya M. Sekizawa K. Okinaga S. Suzuki T. Yamada N.. (1997). Rhinovirus Infection of Primary Cultures of Human Tracheal Epithelium: Role of ICAM-1 and IL-1beta. Am. J. Physiol. 273 (4), L749–L759. doi: 10.1152/ajplung.1997.273.4.L749 9357849

[B45] Van Ewijk B. E. Wolfs T. F. Aerts P. C. Van Kessel K. P. Fleer A. Kimpen J. L.. (2007). RSV Mediates Pseudomonas Aeruginosa Binding to Cystic Fibrosis and Normal Epithelial Cells. Pediatr. Res. 61 (4), 398–403. doi: 10.1203/pdr.0b013e3180332d1c 17515861

[B46] van Wetering S. van der Linden A. C. van Sterkenburg M. A. de Boer W. I. Kuijpers A. L. Schalkwijk J.. (2000). Regulation of SLPI and Elafin Release From Bronchial Epithelial Cells by Neutrophil Defensins. Am. J. Physiol. Lung Cell Mol. Physiol. 278 (1), L51–L58. doi: 10.1152/ajplung.2000.278.1.L51 10645890

[B47] Vareille M. Kieninger E. Edwards M. R. Regamey N. (2011). The Airway Epithelium: Soldier in the Fight Against Respiratory Viruses. Clin. Microbiol. Rev. 24 (1), 210–229. doi: 10.1128/CMR.00014-10 21233513 PMC3021210

[B48] Wang J. Li F. Sun R. Gao X. Wei H. Li L. J.. (2013). Bacterial Colonization Dampens Influenza-Mediated Acute Lung Injury *via* Induction of M2 Alveolar Macrophages. Nat. Commun. 4, 2106. doi: 10.1038/ncomms3106 23820884 PMC3715851

[B49] Wolf A. I. Strauman M. C. Mozdzanowska K. Whittle J. R. Williams K. L. Sharpe A. H.. (2014a). Coinfection With Streptococcus Pneumoniae Modulates the B Cell Response to Influenza Virus. J. Virol. 88 (20), 11995–12005. doi: 10.1128/JVI.01833-14 25100838 PMC4178749

[B50] Wolf A. I. Strauman M. C. Mozdzanowska K. Williams K. L. Osborne L. C. Shen H.. (2014b). Pneumolysin Expression by Streptococcus Pneumoniae Protects Colonized Mice From Influenza Virus-Induced Disease. Virology 462–463, 254–265. doi: 10.1016/j.virol.2014.06.019 PMC4157663 24999050

[B51] Woo T. E. Lim R. Surette M. G. Waddell B. Bowron J. C. Somayaji R.. (2018). Epidemiology and Natural History of Pseudomonas Aeruginosa Airway Infections in non-Cystic Fibrosis Bronchiectasis. ERJ Open Res. 4 (2), 00162–2017. doi: 10.1183/23120541.00162-2017 29930949 PMC6004520

[B52] Yang M. L. Wang C. T. Yang S. J. Leu C. H. Chen S. H. Wu C. L.. (2017). IL-6 Ameliorates Acute Lung Injury in Influenza Virus Infection. Sci. Rep. 7, 43829. doi: 10.1038/srep43829 28262742 PMC5338329

