# Supplementary material for: Pseudomonas aeruginosa Affects Airway Epithelial Response and Barrier Function During Rhinovirus Infection
Source: Front Cell Infect Microbiol. 2022 Feb 21;12:846828. doi: 10.3389/fcimb.2022.846828 (PMC8899922; doi:10.3389/fcimb.2022.846828)
Supplement: Supplementary file 1 [file Table_1.docx]

Supplementary Material

# Supplementary Tables

**Supplementary Table 1.** Donor characteristics for primary cell isolations.

| Donor | Pathology | Age (years) | Gender |
| --- | --- | --- | --- |
| 1 | Emphysema | 58 | f |
| 2 | Emphysema | 49 | f |
| 3 | Emphysema | 57 | f |
| 4 | Cystic fibrosis | 31 | m |
| 5 | Cystic fibrosis | 42 | m |
| 6 | Cystic fibrosis | 33 | f |

**Supplementary Table 2.** Primer and probe sequences for RT-qPCR

| Target | Function | Sequences (5‘-3‘) | Reference |
| --- | --- | --- | --- |
| GAPDH | forward primer | ATTCCACCCATGGCAAATTC | (4) |
|  | reverse primer | CGCTCCTGGAAGATGGTGAT |  |
|  | probe | JOE-CGTTCTCAGCCTTGACGGTGCCA-BHQ1 |  |
| HRV | forward primer | GGTGTGAAGAGCCGCGTG | modified from (5) |
|  | reverse primer | CAAAGTAGTCGGTCCCATCC |  |
|  | probe | FAM-TCCTCCGGCCCCTGAATGTGG-BHQ1 |  |
| IL-6 | forward primer | GGTACATCCTCGACGGCATCT | modified from (6) |
|  | reverse primer | GTGCCTCTTTGCTGCTTTCAC |  |
|  | probe | FAM- TGTTACTCT-ZENQ-TGTTACATGTCTCCTTTCTCAGGGCT-IowaBlackFQ |  |
| CXCL-8  (IL-8) | forward primer | CTGGCCGTGGCTCTCTTG | (7) |
|  | reverse primer | TTAGCACTCCTTGGCAAAACTG |  |
|  | probe | FAM-CCTTCCTGATTTCTGCAGCTCTGTGTGAA-BHQ1 |  |
| Pro- IL-1β | forward primer | CTTCGAGGCACAAGGCACAA | in house assay |
|  | reverse primer | TTCACTGGCGAGCTCAGGTA |  |
|  | probe | FAM-TCTGCCATGGCTGCTTCAGACACT-BHQ1 |  |
| IFN-β | forward primer | CGCCGCATTGACCATCTA | (8) |
|  | reverse primer | TTAGCCAGGAGGTTCTCAACAATAGTCTCA |  |
|  | probe | FAM-TCAGACAAGATTCATCTAGCACTGGCTGGA-BHQ1 |  |
| IFN-λ_1_ | forward primer | GGACGCCTTGGAAGAGTCACT | (8) |
|  | reverse primer | AGAAGCCTCAGGTCCCAATTC |  |
|  | probe | FAM-AGTTGCAGCTCTCCTGTCTTCCCCG-BHQ1 |  |
| FOXJ1 | forward primer | CAACTTCTGCTACTTCCGCC | modified from (9) |
|  | reverse primer | CGAGGCACTTTGATGAAGC |  |
|  | probe | FAM-ACGCAGATCCCACCTGGCAGAA-BHQ1 |  |
| SNTN | forward primer | GTCAGTAGCGACCTAGAGCAC | in house assay |
|  | reverse primer | GCCTGGCCAATTCATTACTGT |  |
|  | probe | FAM-CATCTACTTGATGGAACTGCTGAGGA-BHQ1 |  |
| SCGB3A1 | forward primer | CATAGAGGGCTCCCAGAAGTG | modified from (9) |
|  | reverse primer | CAGCGTCTTGTCCTCAGGTG |  |
|  | probe | FAM- ATGCTCCAGTCTCGGCTCAGC-BHQ1 |  |

# Supplementary Figure


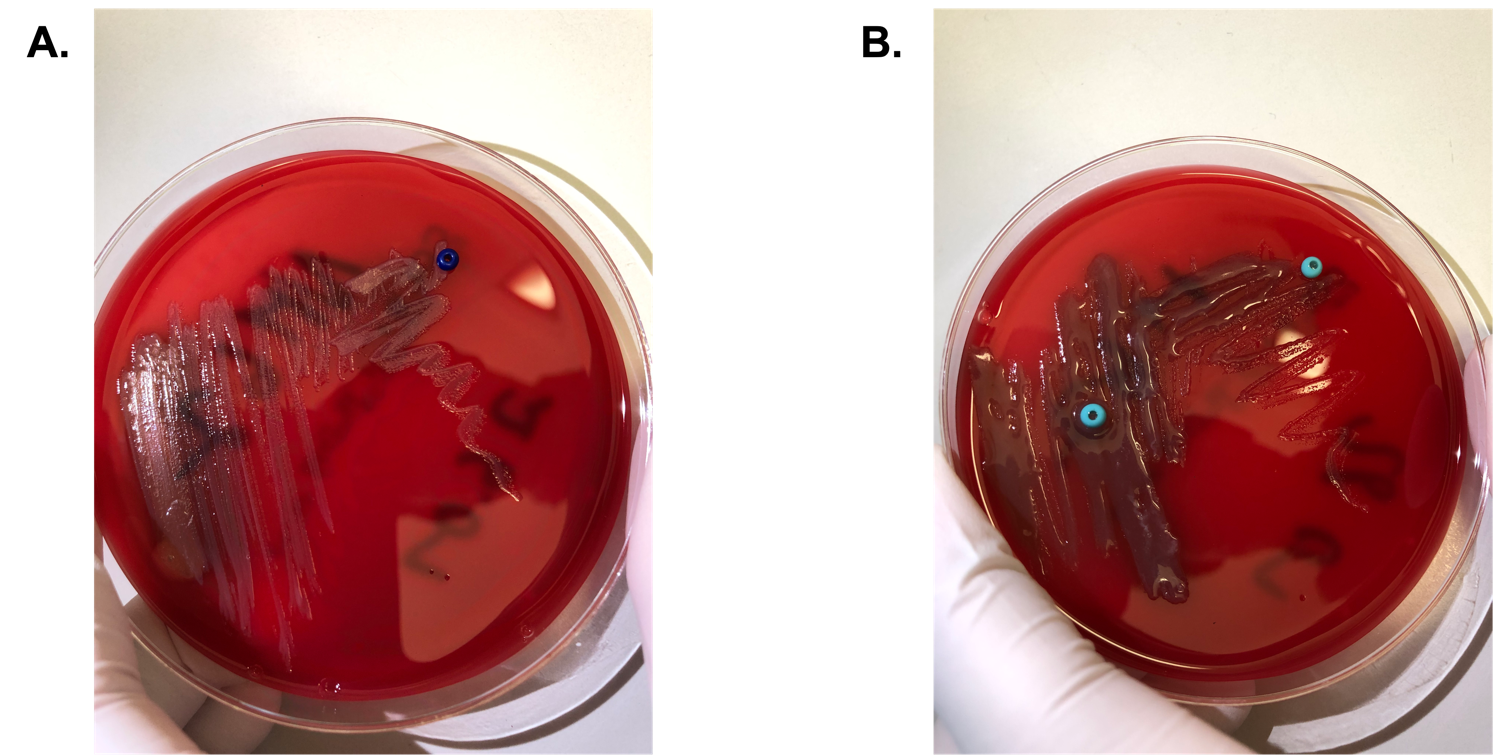


**Supplementary Figure 1:** Overnight cultures of *P. aeruginosa* (PA) clinical isolates used in the study (A) non-mucoid PA (B) mucoid PA.
